# Reducing Results Variance in Lifespan Machines: An Analysis of the Influence of Vibrotaxis on Wild-Type *Caenorhabditis elegans* for the Death Criterion

**DOI:** 10.3390/s20215981

**Published:** 2020-10-22

**Authors:** Joan Carles Puchalt, Pablo E. Layana Castro, Antonio-José Sánchez-Salmerón

**Affiliations:** Instituto de Automática e Informática Industrial, Universitat Politècnica de València, 46022 Valencia, Spain; juapucro@doctor.upv.es (J.C.P.); pablacas@doctor.upv.es (P.E.L.C.)

**Keywords:** image processing, lifespan automation, *C. elegans*

## Abstract

Nowadays, various artificial vision-based machines automate the lifespan assays of *C. elegans*. These automated machines present wider variability in results than manual assays because in the latter worms can be poked one by one to determine whether they are alive or not. Lifespan machines normally use a “dead or alive criterion” based on nematode position or pose changes, without poking worms. However, worms barely move on their last days of life, even though they are still alive. Therefore, a long monitoring period is necessary to observe motility in order to guarantee worms are actually dead, or a stimulus to prompt worm movement is required to reduce the lifespan variability measure. Here, a new automated vibrotaxis-based method for lifespan machines is proposed as a solution to prompt a motion response in all worms cultured on standard Petri plates in order to better distinguish between live and dead individuals. This simple automated method allows the stimulation of all animals through the whole plate at the same time and intensity, increasing the experiment throughput. The experimental results exhibited improved live-worm detection using this method, and most live nematodes (>93%) reacted to the vibration stimulus. This method increased machine sensitivity by decreasing results variance by approximately one half (from ±1 individual error per plate to ±0.6) and error in lifespan curve was reduced as well (from 2.6% to 1.2%).

## 1. Introduction

Lifespan assays in *C. elegans* has become one of the most widespread research trial models [1,2]. Assay procedure is based on daily nematode survival counts in large populations. Traditionally, this process has been conducted manually by experts, whose ability to discern whether a worm is dead or alive entails several issues, such as [1]. The dead or alive criterion is commonly inferred from *C. elegans* movement, which categorizes the worm as alive if movement is detected, and dead otherwise. However, this is complicated due to animal slowness in accordance with its ageing, which ends when worms fail to move at all during inspections. This is why nematodes are mechanically stimulated by an expert applying pick stimulation to confirm death [3]. This task, which has to be done to each worm successively, is both arduous and laborious. Given the fact that lifespan assays are labour-intensive, repetitive and time-consuming, various devices have been developed for assay automation to improve *C. elegans* assay throughput [4,5,6]. These lifespan machines are generally based on computer vision, and they attempt to emulate manual inspection procedures by interpreting sequences of captured images. Despites these advances, lifespan machines must overcome challenges concerning the detection of motility on the last days of worm life because they become very lethargic, requiring *C. elegans* monitoring over long time spans in order to observe any motion. This leads to a series of complications, such as memory for image storage, computational load, inspection times, etc. The issue related to lack of motion in manual inspection has been solved by mechanical stimulation with a platinum wire pick but, to date, lifespan evaluation devices have proved ineffective. There are high-throughput handling mechanisms that can help to stimulate the worms. Automating a platinum wire pick to individually tap each worm is an expensive and complex solution, which is also slow to execute [7]. Other kinds of mechanical stimulation exist [8,9,10,11], which are non-localised, such as vibration [12,13,14,15]. Vibration is able to induce a withdrawal response by stimulating *C. elegans*; namely, vibrotaxis, whereby the worm responds to mechanical vibrations. Tapping causes a mechanical wave that propagates through the medium so, in essence, tapping on a Petri plate is a vibrational source. Mechanical vibration has the advantage of being transmitted through the medium to the whole sample and can, thus, achieve large-scale stimulation, and develop a simpler, more economical vibration system. After an in-depth state-of-the-art study, no reference was found in which vibration stimulation has been used as a method to verify nematode life or death in lifespan machines. It has been employed for other purposes, such as modelling nematode mechanosensory neurons [16], modelling withdrawal response behaviour [8], identifying genes, and worm memory and habituation [17]. Some examples of techniques are available whose implementation involves no kind of vibrator system. MWT [18] resorts to a solenoid tapper to tap plates to study memory and habituation. This machine is also used to observe behaviours like chemotaxis and food preference. To demonstrate that sensory modulation is integrated at many levels [12], a dual cone speaker was utilised. This device allowed the configuration of vibration parameters which, in turn, enabled tests to study behaviour and memory consisting of a sound piezoelectric sheet speaker [15]. Ultrasound devices are employed to reveal the molecular mechanisms of ultrasound neuro-modulation [19]. Besides mechanical stimulation, phototaxis can also be used as a stimulation method. Worm exposure to light induces to withdrawal responses [20,21,22,23,24], principally using a blue-light wavelength. Nevertheless, high intensity light is required to stimulate worms, which affects their lifespan and can even kill them [25].

Here, we present a new method based on vibration to stimulate *C. elegans* in Petri plates for lifespan assays, to confirm whether worms are dead or alive. This new method permits the monitoring of nematodes in plates and can be adapted to any automatic inspection device. This method provides more robust detection of worm on their last days of life, during which they hardly move, and reduces lifespan results variance by approximately one half. It is easily automatable and stimulates every worm at the same time, achieving a very high animal response ratio (>93%). In addition, there is no statistically significant indication that vibration affects the nematode’s life expectancy.

## 2. Materials and Methods

We used a system composed by a lifespan machine (lighting and vision subsystems) and our proposed vibration subsystem. This system is an improved sensor to calculate lifespan automatically, which enabled us to compare lifespan results with and without vibration conditions. Three experiments were performed to analyse several effects. The vibration timing experiment to study worms’ responses (vibrotaxis) to different vibration stimuli. The habituation experiment to analyse the worm inhibition to repetitive stimuli. And Lifespan error experiment to study the improvement in live worm detection by comparing the detection of two different lifespan conditions.

### 2.1. Lifespan Machine

**Figure 1:**
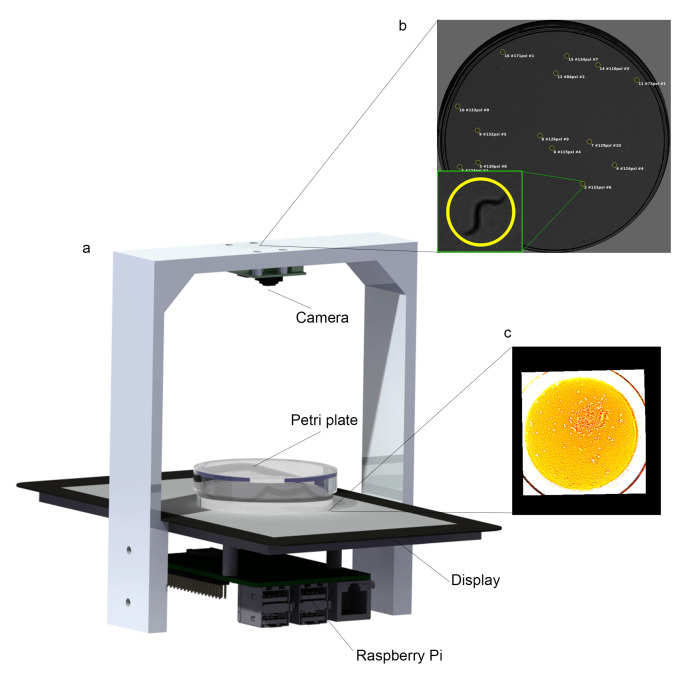
Scheme (**a**) Lifespan machine used. (**b**) A Petri plate image, with a zoom of an example worm. (**c**) Illumination pattern example which is drawn on display.

We worked with an automatic lifespan machine [26] and the typical microscope configuration as shown in Figure 1a, where the camera is placed above (Raspberry Pi camera rev 1.3), low-intensity lighting (Raspberry Pi 7” display) is positioned below (backlight), and Petri plates are placed between them (camera is to 77.5 mm from the object). The automatic camera settings are disabled, and the shutterspeed and brightness values are set at 100,000 μs and 25 respectively. It employs one camera to take images of the entire Petri plate (55 mm), it gives about 30 μm/pxl. Nevertheless, there are small dark zones in the wall areas where shadows appear, which represents less than 5%. Consequently, there is a small probability that a worm may be hidden from view. This method is based on active vision, which is used to control light intensity making every image pixel reach a given level of intensity. The reasons for this controlled intensity are: (1) to improve image quality and (2) to stress worms as little as possible due to high light intensity (phototaxis effect). The basic procedure consists in taking an image (Figure 1b) and applying image transformation to obtain an illumination pattern (Figure 1c), which is drawn on the display. More details are to be found in [26].

The experiments run on this machine involve two steps: the first step consists of image sequence acquisition once a day. The second step involves offline processing of image sequences to extract a survival curve for each condition. In this machine, the dead or alive criterion and image processing are defined by the method in [27]. A sequence of 30 images per plate is taken every day at 1 fps. From this sequence information is extracted about nematode motion detection, when a pixel value changes. This consisted of classifying pixels by their signature templates. This involved pixel segmentation per image by taking a fixed 33 grey intensity threshold and avoiding any manual threshold adjustment. This fixed threshold segmentation procedure was possible because the background pixels were controlled as being close to grey level 48 by an active lighting system. If all the values were black, this pixel was classified as ‘constant dark’. If all the values were white, it was classified as ‘constant white’. ‘Noisy pixels’ and ‘pixels in motion’ presented different patterns switching between black and white. Specifically, ‘noisy pixels’ presented a higher frequency of changes than pixels in motion. Standard computer vision algorithms, such as tracking and images alignment, allows us to automatically obtain lifespan curves from the image sequences that were captured once daily throughout the assay.

### 2.2. Vibration Mechanism

**Figure 2:**
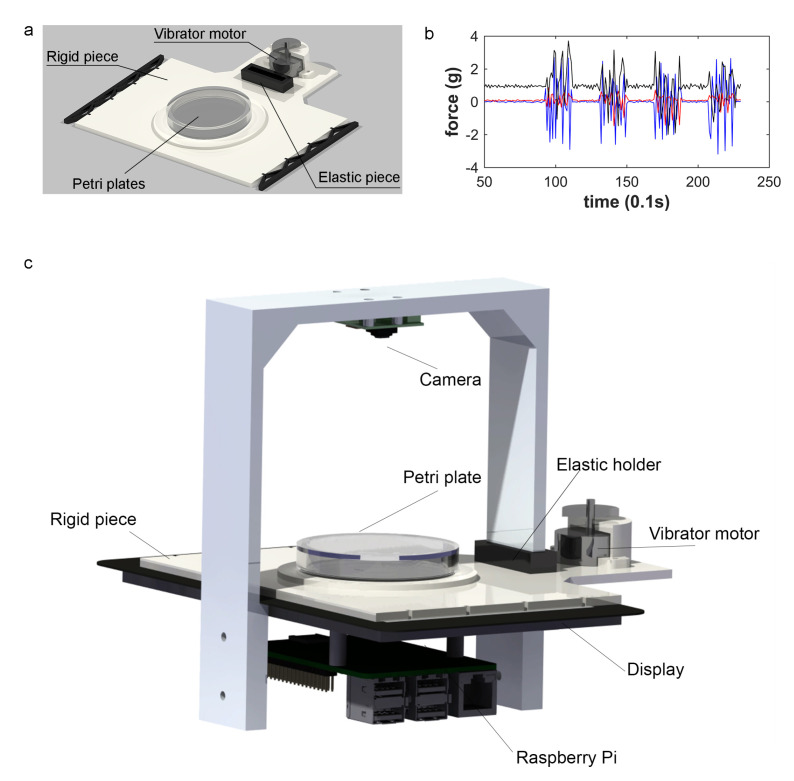
Vibrator system (**a**) Vibration system parts. (**b**) In our study, a Petri plate mounted on the rigid component was subjected to vibrations of between 2 g and 3 g on the X axis and the Z axis, and between 1 g and 2 g on the Y axis, when the vibrator motor was fed 12 V for 3 s time periods. The measurements were taken by an Inertial Measurement Unit (IMU) GY-521, recording the values shown in (**b**) (pallet vibration intensity). (**c**) Assembled altogether: vibration system adapted and the lifespan machine used.

We have designed and developed a vibration system (Figure 2a) to be installed in the automated lifespan system previously cited [26], and schematically represented in Figure 2c. The vibration method is based on a vibrator motor and, therefore, this simple system can be redesigned and adapted to other lifespan machines. The structure was produced with a 3D printer, composed of both rigid and elastic pieces. The elastic pieces restrain the rigid ones in the equilibrium position, which allows for displacement due to deformation of the elastic part, which returns to the initial position once vibration stops. Figure 2a shows that the rigid component (white coloured material) is the main structure onto which the vibrator motor is fixed (grey actuator on figure), and the elastic support and the Petri plate are thereto attached. The motor provides the source of vibration, transmitted through the rigid structure to the Petri plate. Vibrations (Figure 2b) are produced when the vibrator motor spans an asymmetric mass. Therefore, the mechanical parameters are invariant, except for angular velocity (frequency), which denotes that vibration frequency and intensity are dependent variables because they depend on angular velocity. Therefore in this case, the controllable variables are angular speed and application time. The guidelines to build this system and the assembly description can be found in this repository (https://github.com/JCPuchalt/vibrotaxis).

### 2.3. Sample Design

Nematodes were provided by the Cell Biology Laboratory at ADM Nutrition/Biopolis SL/Archer Daniels Midland. They were maintained by following standard methods [28]. A *C. elegans* wild-type strain culture N2 was prepared and all nematodes were age synchronised and pipetted onto solid NGM in 55 mm Petri plates, and fed *Escherichia. Coli* strain OP50. On the first day of worm adulthood, the plates were stored in an incubator in the dark. Temperature was maintained at 20 °C. FUdR (0.2 mM) was added to plates to sterilise worms [29] and to, thus, ensure a constant number of individuals, and fungizone (1 μg/mL) was added to prevent fungal contaminations. FUdR alters lifespan, and therefore a control condition is used with which to compare all conditions. Young adult worms (15 per plate) were used for the first day of lifespan experiments.

### 2.4. Experimental Design

Three experiments were designed. For all the experiments, the vibrator system was mounted on the automated lifespan machine [26]. These components were fitted inside an incubator to maintain Petri plate temperature constant, and to prevent external light from reaching them. For all the experiments, the data on the contaminated dishes were censored. For the vibration assays, the prototype rested on four silicone supports so that no vibration was transmitted to neighbouring devices. These vibration conditions were subjected to vibration for *t* s, followed by a sequence of images that was saved for 30 s. These conditions were named *V* with subscript *d* (the day they started undergoing vibration) and subscript *t* (vibration duration in seconds). Every vibration condition has two plates, each one with 15 worms (n=30). The conditions with no stimulus were also captured for 30 s, and their nomenclature was NV (No Vibration).

#### 2.4.1. Methodology before and after the Vibration Conditions

**Figure 3:**
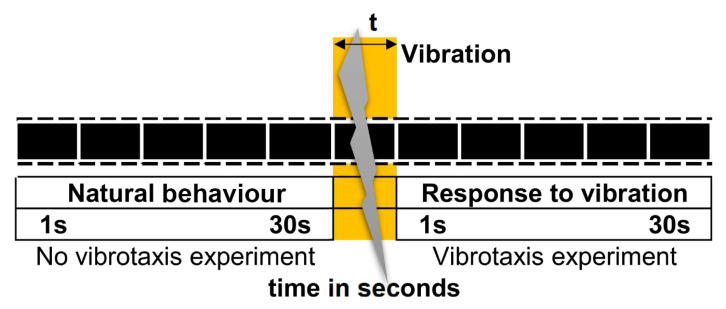
Before and after the vibration methodology. Image sequences: a continuous image recording at 1 fps for 30 s (natural behaviour), followed by vibration applied for *t* s, and finally another 30 s sequence when worm reaction was recorded.

The Vconditions of experiments 1 and 2 were based on worm movement comparison made between, before and after vibration, in addition to its reaction and detection. Thus, it was necessary to acquire images of two consecutive sequences (Figure 3). The first sequence consisted of 30 images at 1 fps, which was before vibration when natural worm behaviour could be recorded. As soon as the first sequence finished, vibration was applied, followed by a second sequence (also 30 images) when worm response to vibration was analysed.

#### 2.4.2. Experiment 1: Vibration Timing Experiment

**Figure 4:**
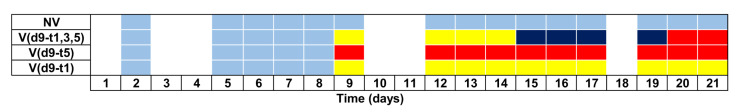
Timeline Experiment 1: Vibration timing/response experiment. White squares denote days with no data acquisition, light blue squares were inspected without vibration (*t* = 0 s), yellow squares show the worms stimulated for 1 s (*t* = 1 s), dark blue ones for 3 s (*t* = 3 s) and red ones for 5 s (*t* = 5 s).

The aim of this experiment was to study worms’ responses (vibrotaxis) to different vibration stimuli. Three different vibration timings were applied in this experiment by defining three conditions, $V_{d_{9}-t_{1}}$, $V_{d_{9}-t_{5}}$ and $V_{d_{9}-t_{1,3,5}}$, as shown in Figure 4. The light blue squares represent zero vibration and the white squares mean no data were collected. The other squares show that a 1 s stimulus was applied (yellow squares) to the $V_{d_{9}-t_{1}}$ condition. For the red squares, a 5 s stimulus was applied to the $V_{d_{9}-t_{5}}$ condition. The $V_{d_{9}-t_{1,3,5}}$ condition was subjected to stimulus lasting 1 s (days 9, 12, 13 and 14), 3 s (days 15, 16, 17 and 19) and 5 s (days 20 and 21). The sample size of the three conditions was about 140 worms per condition with nine plates per condition.

By using the vibration methodology before and after, as shown in Figure 3, this experiment enabled comparisons to be made of the amount of movement variation for these conditions, the percentage of worms that responded to vibration (response index to the stimulus) and the number of live worms detected.

#### 2.4.3. Experiment 2: Habituation Experiment

**Figure 5:**
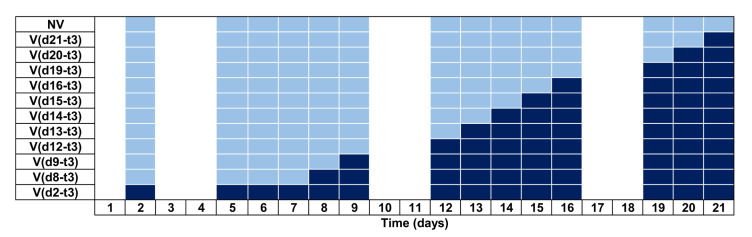
Timeline Experiment: Habituation experiment. The white squares are days with no data acquisition, the light blue squares were inspected without vibration (*t* = 0 s) and the dark blue ones for 3 s (*t* = 3). It represents the vibration application (dark blue squares) during the lifespan per condition.

The habituation effect was analysed by changing the number of times that vibration was applied. A 3 s long vibration was applied once daily, starting on a specific day “d” that depended on the condition, and continued until the end of the nematode lifespan (see Figure 5). The dark blue squares represent the stimulus application days for each condition depicting before and after the vibration method (Figure 3). The light blue squares denote the data acquisition without any vibration in order to avoid the habituation effect. The $V_{d_{2}-t_{3}}$ condition obviously achieved the highest levels of stimulation (from day 2 to day 21) while no vibration was applied for the NV condition (No Vibration). Each condition consisted of samples with two Petri plates containing approximately 15 worms (n=30), except for $V_{d_{2}-t_{3}}$ with 10 Petri plates n=174 and the NV condition with n=170.

#### 2.4.4. Experiment 3: Lifespan Error Experiment

**Figure 6:**
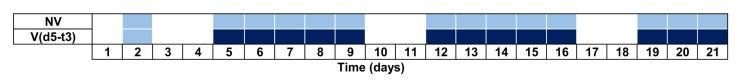
Timeline Experiment 3: Lifespan error experiment. White squares denote no data acquisition. Light blue squares depict data acquisition, but no stimulus. Dark blue ones represent data acquisition with a 3 s stimulus.

This experiment aimed to study the improvement in live worm detection by comparing the detection of two different lifespan conditions (Figure 6).

**Condition NV,** no vibration was applied, as shown in Figure 6 (NVcondition), whose measures were taken once daily, except at weekends. Two replications were used: $n_{1}=113$ and $n_{2}=124$. Condition $V(d_{5}-t_{3})$. This was done by applying vibration *t* = 3 s immediately before the 30 s image capture (Vcondition), which is the time required for Petri plate inspection. The sample size was n=114 individuals and days with vibration were 5, 6, 7, 8, 9, 12, 13, 14, 15, 16, 19, 20 and 21.

### 2.5. Ground-Truth Data for the Validation Method

For each experiment, the lifespan curves were counted manually by an expert to obtain reference nematode survival values (ground-truth). The followed technique was the same as that for automatic counting (no change in shape and position on the current day and the previous day), but with human supervision by inspecting the machine-captured images. If the number of worms detected was higher on one day than on the previous day, we inferred that worms had remained hidden from view and, therefore, the value was regressively rectified. For experiments 2 and 3, this manual count was done by analysing the captured sequence of 30 images. However for experiment 1, this manual count was obtained by inspecting each plate on three occasions and at three different times by considering the highest of the three values. Therefore, this approximation provides a more accurate value than other experiments because this procedure detects more hidden worms.

## 3. Results

### 3.1. Vibrotaxis Analysis

**Figure 7:**
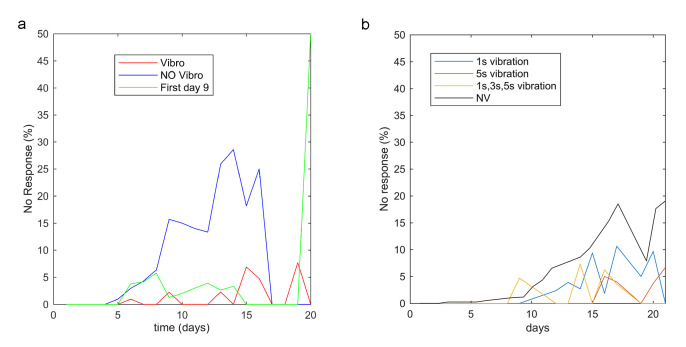
No movement detection. (**a**) In experiment 2 (habituation experiment), few animals were left in the three samples on the last days (2–4), due to nematode death. At such low survival rates, the non-response ratios were variable as of day 18. On day 20 of “first 9 days of the stimulation conditions” (green curve), the non-response ratio was 50% because there were only two worms left. (**b**) Experiment 1, the blue condition shows the no-response percentage after applying a 1 s vibration, the red condition applied a 5 s vibration, the orange one lasted 1 s [on days 9, 12, 13 and 14], 3 s [days 15, 16, 17 and 19] and 5 s [days 20 and 21], while the grey one denotes the no-vibration condition.

On hypothesizing that vibration-based stimulation could improve lifespan evaluation, it was important to prove whether or not nematodes reacted to vibrotaxis. The no-response ratios corresponded to live worms with no detected movement, as shown in Figure 7 for certain conditions taken from experiments 1 (Figure 7b) and 2 (Figure 7a). In experiment 2 (Figure 7a), on day 9 a significant worm percentage (15%) was obtained for the NVcondition, which began with a lack of movement recorded during the 30 s inspection for signs of ageing. This percentage continued to increase throughout the animals’ lifetime to reach 29% no detectable movement. The worms subjected to vibration (Vconditions, 3 s) reacted by moving, mostly throughout their whole life. The no-response rates significantly increased only on the last 2 or 3 days of their lifetime. Thus by applying a 3 s vibration stimulated worm movement with the following results: 99% in week 1, 98% in week 2 and 93% in week 3 (Figure 7a).

### 3.2. Habituation Analysis

**Figure 8:**
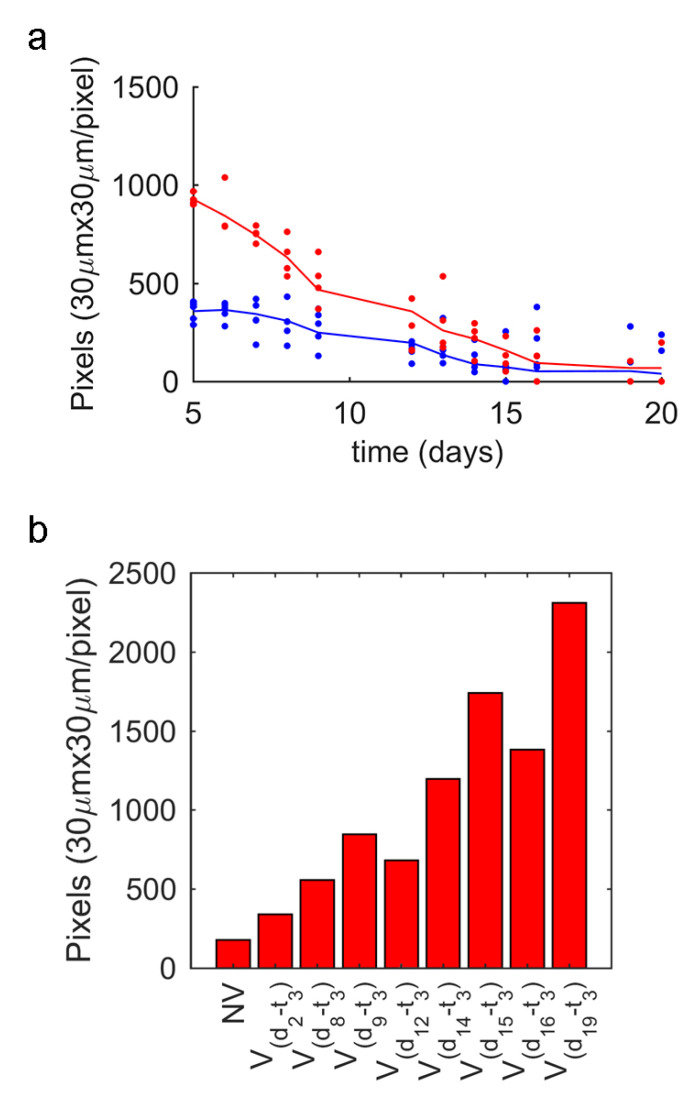
Ageing effect. (**a**) Amount of movement (area in pixels) in time, blue line denotes the NVcondition and the red line is $V_{d_{2}-t_{3}}$ (vibration started on day 2); (**b**) This figure shows the same as the previous one, but with details of day 19. Each bar is a condition (left to right) to represent from the lowest stimulus $V(d_{2}-t_{3})$ to the highest $V(d_{19}-t_{3})$.

There was a memory and habituation effect, as shown in Figure 7a,b, whereby worms became used to stimulation and, consequently, to respond less to it. Because of habituation, worms displayed less reaction sensitivity (Figure 7a) and less intensity (Figure 8a) and, therefore, the number of nematodes that reacted decreased and those that reacted displayed fewer movements. The mean movement measured 500 pixels^2^ in 30 s and the maximum was 1200 pixel^2^ (Figure 8a), the worm width is 3 pixels, thus equivalents in linear millimetres were 0.14 mm/s and 0.34 mm/s respectively, which corresponds to other studies targeting this phenotype [4,18,30,31]. The habituation effect was observed on the last 2 or 3 days of worms’ lives (danger awareness vs. ageing) when comparing two groups (Figure 7a): the NVcondition had a 71% response (blue curve) and the Vcondition had a 93% response (red curve). The third group (green curve) was submitted to less stimulation (starting on day 9), which improved its response on the last days with a 100% response between 15 and 19 days for the same vibration time (3 s) versus the condition stimulated from day 2. Due to this finding, it seemed better to seek a strategy whereby vibration was not applied from day 1, but rather from the day on which a significant percentage of live *C. elegans* started to go undetected by movement. The first day could be set between days 5 and 9 for the wild-type strain, whose no-response rates were between 2% and 15%. In addition, changing vibration parameters may alleviate habituation, as indicated by the data taken from experiment 1 (Figure 7b), whose results show that vibrotaxis increased by changing the vibration time every 5 days (from 1 s, 3 s to 5 s). On the days when vibration timing changed (days 15 and 20), the worm response was 100% and the response average increased. In general, high magnitudes of vibration times (5 s) also seemed to reduce habituation to the stimulus for a longer time, but adaptation was finally successful (days 20 and 21; see Figure 7b). It is also important not to overstress nematodes. Therefore, we considered it appropriate to choose short vibration times and to reduce application days. According to previous deductions, and as a starting point for further studies, it seemed reasonable to choose the first day to be somewhere between days 5 and 9 to begin the stimulus and apply a vibration time of at least 3 s.

We can observe the ageing effect (Figure 8a), and how the amount of movement reduced for conditions NV and $V_{d_{2}-t_{3}}$ ($V_{d_{2}-t_{3}}$ shown after stimulation). In a detailed view (Figure 8b), the stimulation habituation effect can be seen after being analysed. Subsequent to vibration, Figure 8b shows a trend by which worms were stimulated on a greater number of days; these nematodes moved less than those receiving the stimulus for fewer days. A reduction in movement can be problematic because if a nematode does not move enough at a specific image resolution, the software may not be able to detect it during inspection.

### 3.3. Error Variability Analysis

After considering memory, habituation and ageing issues, in order to verify that this new method could improve the no-response ratios of an automated vision machine, we compared the detection error to two factors (stimulation versus no stimulation) with several of the samples taken from experiments 1, 2 and 3. Thus for the Vcondition, in the lifespan error experiment (experiment 3) we attempted to maximize the *C. elegans* vibrotaxis effect by selecting the parameters deduced from the vibration timing experiment (experiment 1) and the habituation experiment (experiment 2), according to which we chose both parameters: (1) a 3 s vibration for stimulation and (2) stimulus applied from day 5. The NVcondition of this experiment was performed without vibration. To compare the total error of the lifespan curves for both conditions, we compared them for the same sample size. Thus two replications were studied for this condition. The error per day ($e_{\left(k\right)}$) is the difference between manual lifespan curve (SM) and the automatic lifespan (SA) for a specific day (*k*) (Equation (1)) of the sum of all Petri plates for one condition. The total error ($e_{T}$) (Equation (2)) is the mean of all $e_{\left(k\right)}$, for those days with data acquisition ($N_{k}$).

(1) $$e_{\left(k\right)}=\left|SM_{\left(k\right)}-SA_{\left(k\right)}\right|$$

(2) $$e_{T}=\frac{\sum_{k}^{N_{k}}e_{\left(k\right)}}{N_{k}}$$

Obviously, when a more accurate approach in detection is achieved, the sum of all errors (Equation (2)) tends to zero, therefore the error is analysed at Petri-plate level. The error per plate (Equation (3)) is the difference between ground truth per plate ($X_{p}$) and measured value per plate ($x_{p}$). The mean error per plate (Equation (4)) is the mean of the error of all plates ($N_{p}$) for a certain condition. Finally, the standard deviation of the plates errors for each condition is defined in (Equation (5)).

(3) $$e_{P_{\left(p\right)}}=x_{\left(p\right)}-X_{\left(p\right)}$$

(4) $$e_{P}=\frac{\sum_{p=1}^{N_{p}}e_{P_{\left(p\right)}}}{N_{p}}$$

(5) $$sigma=\sqrt{\frac{\sum_{p=1}^{N_{p}}{e_{P_{\left(p\right)}}}^{2}}{N_{p}-1}}$$

**Figure 9:**
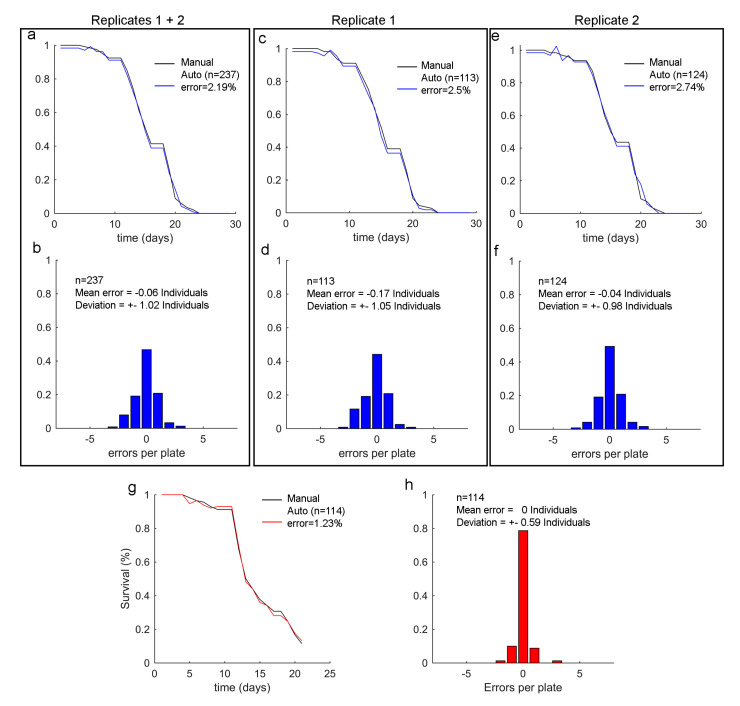
Lifespan results of experiment 3. (**a**–**f**) belong to the NVcondition. (**a**) is the survival curve as a percentage per one for all the replications. (**b**) is the error distribution per plate for all the replications. (**c**,**d**) are replication 1.(**e**,**f**) are replication 2. (**g**,**h**) belongs to vibration condition $V(d_{5}-t_{3})$.

The results for this condition gave (Figure 9c,d) n=113 individuals, a total error ($e_{T}$) of 2.5%, a mean error per plate ($e_{P}$) of −0.17 individuals and a deviation (sigma) ±1.05 for replication 1. For replication 2 (Figure 9e,f), sample size was 124 individuals, with a total error of 2.7%, a mean error per plate of 0.04 individuals and deviation ±0.98 individuals were obtained. For both replications (Figure 9a,b), sample size was 237 individuals, with a total error of 2.19%, a mean error per plate of −0.06 individuals and deviation ±1.02. Therefore for the NVcondition, the standard deviation error per plate was about ±1 individuals. The Vcondition (Figure 9g,h) had n=114 individuals, a total error of 1.23%, a mean error per plate of 0 individuals and deviation ±0.59 individuals. According to these experimental results, when stimulation lasted 3 s and started on day 5, the total error ($e_{T}$) lowered for similar sample sizes of about 120 worms, from approximately 2.5% to 1.2%, with an uncertainty reduction from 1.0 individuals to 0.6 individuals (standard deviation of error per plate, sigma). Moreover, we found a statistically significant difference between both variances of error, with *p*-Value ≈ 0. This demonstrated a reduced uncertainty.

As previously mentioned, experiments 1 and 2 were performed to define vibrations *d* and *t*. Nevertheless having several sample groups, for which plates were inspected before and after applying the stimulus, made it possible to study the improvement in the margin of error during detection, although these experiments were not comparable because some conditions had changed. Based on these experiments, it was possible to increase the assay number to develop a better approach, in which an improvement in detection was observed when vibration was applied. In all cases, fewer errors were detected in the samples subjected to vibrations than the non-stimulated samples, reducing errors by 50% in some cases thanks to the vibrotaxis-associated movement.

**Figure 10:**
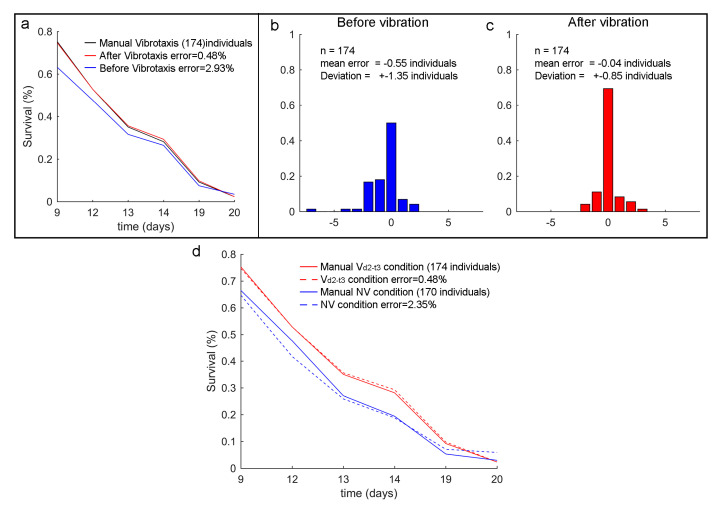
The lifespan results of experiment 2. (**a**) are the survival curves of the $V_{d_{2}-t_{3}}$ condition before and after vibration. (**b**) is the error histogram before vibration, (**c**) is the error histogram after vibration. (**d**) is experiment 2 survival curve for two samples: one was subjected to vibration (the previous $V_{d_{2}-t_{3}}$ condition) and one was not (NVcondition). It is possible to compare the errors of both methods.

For the habituation experiment (experiment 2), only two of the eleven conditions were compared: the least exposed to vibration (NV) and the most exposed ($V_{d_{2}-t_{3}}$). Both had similar sample sizes, 170 and 174, respectively. The error results (Figure 10) showed how the Vcondition before vibration (Figure 10b) had a ±1.35 deviation per plate, which changed to ±0.85 (Figure 10c) after deviation. When comparing the survival curves (Figure 10a), the total error went from 2.9% to 0.48%. In order to observe possible differences for the NVconditions, Figure 10d shows that the error was 2.35%, which was similar to the Vcondition before vibration (2.9% error).

**Figure 11:**
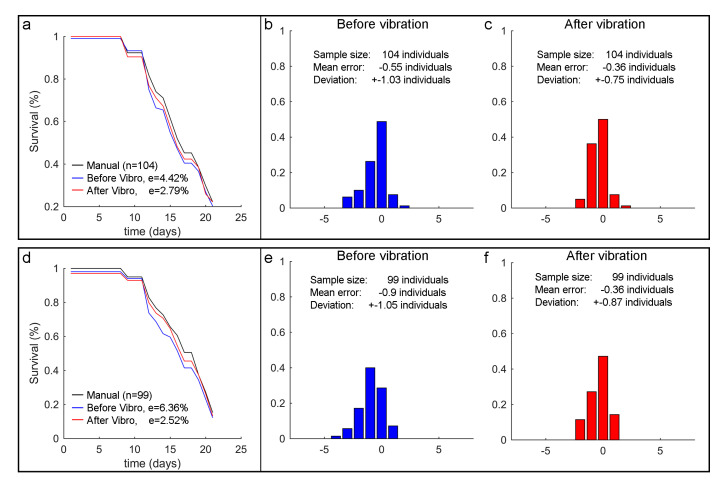
Lifespan results of experiment 1. (**a**–**c**) are the condition $V_{d_{9}-t_{1}}$ sample (subjected to a 1 s vibration from day 9). This represents an error before and after vibration. (**d**–**f**) are for the condition $V_{d_{9}-t_{5}}$ sample (subjected to a 5 s vibration from day 9).

In experiment 1 (Figure 11), manual measurements were taken during three periods; these three measurements gave rise to different results because some worms may have been hidden by wall shadows cast in some areas. We selected the maximum values of these three measurement periods. Hence, in this experiment, the manual curve was more accurate. In this scenario the actual worm number was slightly higher than that detected by a single daily measurement. The reason for this was that the errors in experiment 1 were slightly higher (Figure 11). Similarly to the previous assay results, the error also reduced with vibration for the 1 s stimulation, before (Figure 11b), with deviation ±1.03 individuals per plate, and after (Figure 11c) this value was ±0.75, while the errors in curves were 4.42% and 2.79%, respectively (Figure 11a). For the 5 s vibration, the deviation error before the stimulus was ±1.05 (Figure 11e), the total error was 6.36% (Figure 11d) and after vibration (Figure 11f) there were ±0.87 individuals error per plate and a 2.52% total error.

The previous results would seem to indicate that the larger the sample size, the nearer the mean error would move towards zero, although some uncertainty remained. With this method, uncertainty improved by reducing standard deviation. Thus, reducing the total error also diminished the sample size for the same error, and in such a way that costs and times were cut and accuracy increased.

Worm movement began to decrease significantly between days 5 and 9, in fact some individuals even remained motionless during inspection time. Thus, it is logical to start vibrotaxis and its analysis from these days onwards (Figure 7a), because this new method improved the error from these days on. Furthermore, the most interesting range for the survival study was approximately around the mean lifespan time, which was on about day 14 in the wild-strain (N2).

### 3.4. Vibrotaxis Effect on the Lifespan Analysis

**Figure 12:**
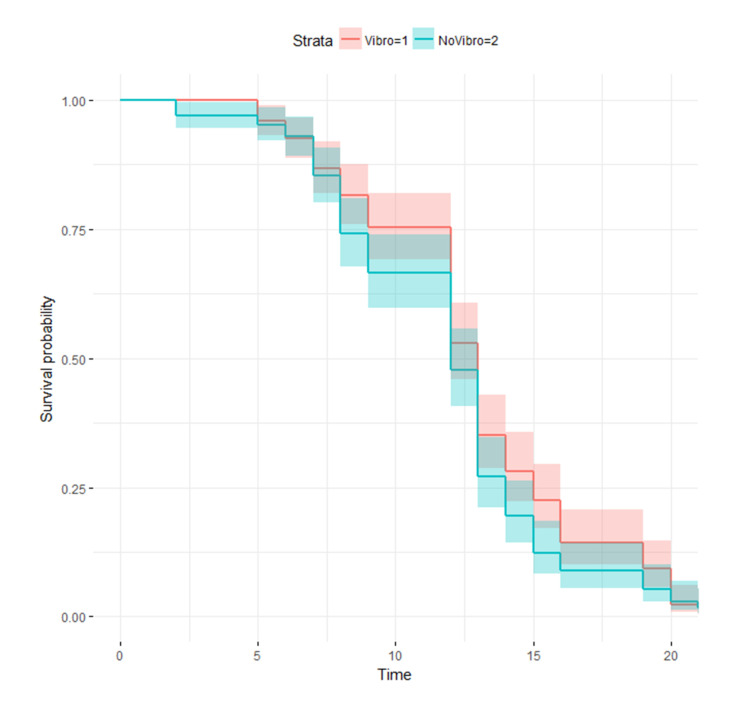
Vibrotaxis effect in lifespan. Kaplan-Meier survival curves for NV (blue) and $V_{d_{2}-t_{3}}$ (red) conditions of experiment 2.

Finally, we studied the survival curves between conditions NV and $V_{d_{2}-t_{3}}$ (extreme cases) of the habituation experiment to verify that vibration did not affect nematode life expectancy. These curves corresponded to the manually recorded ones in experiment 2 ($n_{NV}=170$ and $n_{V(d_{2}-t_{3})}=174$), which were used because manual counting was our ground-truth. Through the Log-rank test and the Cox proportional hazards regression model, we observed there were no statistically significant differences between them, with *p*-values of 0.06 and 0.105, respectively, as shown in Figure 12, where both conditions are drawn with the Kaplan-Meier estimator. Thus it can be inferred that statistically, vibration did not affect the wild-type strain lifespan. According to the data obtained and the vibration type applied during lifespan, vibrotaxis appeared not to affect the life expectancy of *C. elegans*. Vibration lasted only 3 s each day with an acceleration of about a 4 g peak. Consequently, applying this new method without altering the results due to the stress applied is a fundamental factor.

## 4. Conclusions

The new method reported here aims to improve live worm detection, which is achieved by worm motion response rates of more than 93% (depending on the case). This fact demonstrates our technique is an effective stimulation method and, in some cases, the sensitivity for lifespan of the automated systems under the same conditions even doubled (from 2.6% to 1.2%), and the error variance per plate reduced by half (from ±1 individual to ±0.6). According to the findings obtained in our experiments, vibration did not statistically alter *C. elegans* life expectancy (wild-type strain).

Therefore, we have designed a new simple automated inspection system which increases throughput over manual methods and improves sensitivity in detection for automated methods. This method avoids manually stimulating worms one by one, which releases the expert from these repetitive tasks. Therefore, it is possible to reduce the sample size for the same error value, which means reducing both time and costs. In addition, worms respond to vibration quickly. Hence sufficient motion leads to more rapid motion detection, which in turn allows for a shorter monitoring period.

In our experiments, we also found that habituation reduced movement. The habituation effect is caused by the worms’ ability to remember past non-threatening situations and recognise similar ones, although high vibration makes this habituation more difficult. An overstimulation can be a limitation to improve lifespan results with the proposed method. Consequently stimulation needs to be as infrequent as possible. Hence stimulation should be initiated between day 5 and day 9 throughout the remainder of their lives, a time at which the N2 nematodes began to become lethargic, whereupon attempts were made to stimulate motion.

The improvement afforded by our method also depends on the cleaning and condensation conditions because errors may increase with a critical degree of these factors. However, smooth condensation problems can be alleviated by our technique given both the position change and/or image grey level variation.

If we consider that the observed movement responses caused by vibration in the wild-type strain were significant, the proposed method is highly promising and can be applied to other strains. Furthermore, this method could be applied to the phenotyping of strains by undertaking studies of certain mechanosensory problems, which we will carry out in a forthcoming work.
